# Petrified in a Pit

**Published:** 1891-08

**Authors:** 


### PETRIFIED IN A PIT.

Mary Ann Grier disappeared from her father's home, two miles south of Wanatah, Ind., nearly forty years ago. A few days ago, so the story runs, her body was recovered in an abandoned bog iron ore pit, without one vestige of change from the appearance it had known in life. The last shred of clothing was long ago destroyed by the action of the water in which she had met her death, but the same chemicals which removed the garments preserved the flesh. Not only is the contour of the form perfect as in life, but even the color has remained unchanged. The arms and shoulders are as white as marble, the hands are brown, and one of them still bears the stains of the berries with which she was working the afternoon of her disappearance. The body was found in an old pit whence ore had been at one time removed ; but time coated the surface of the water over with a growth of grass that year after year became more firm until the body was entirely buried beneath. When the body was first found it was believed to have been that of one who had recently died, but when struck it emitted a metallic ring, and then it was known that the body was petrified. After a time the aged father of Mary Ann identified the remains as those of his daughter, and her two brothers also confirmed the identity. It is believed the girl fell into the pit when she disappeared nearly forty years ago, drawing down upon her a ledge of the loose soil that completely buried her. The water in the pit mixed with the iron ore produced a solution that petrified the body.

